# Stationäre chirurgische Versorgung in Großschadenslagen und Katastrophen – aktuelle Behandlungskapazitäten in Abhängigkeit von Alarmierungsstatus des Krankenhauses und Versorgungskonzept

**DOI:** 10.1007/s00104-023-01975-x

**Published:** 2023-11-10

**Authors:** Axel Franke, Wolfgang Lehmann, Thomas Wurmb

**Affiliations:** 1 Sektion Unfallchirurgie, Klinik für Unfallchirurgie, Orthopädie, Hand- und Rekonstruktive Chirurgie, Verbrennungsmedizin, BundeswehrZentralkrankenhaus Koblenz, Rübenacher Str. 170, 56072 Koblenz, Deutschland; 2 Klinik für Unfallchirurgie, Orthopädie und Plastische Chirurgie, Universitätsmedizin Göttingen, Robert-Koch-Str. 40, 37099 Göttingen, Deutschland; 3 Klinik und Poliklinik für Anästhesiologie, Intensivmedizin, Notfallmedizin und Schmerztherapie, Sektion Notfall- und Katastrophenmedizin, Universitätsklinikum Würzburg, Würzburg, Deutschland

**Keywords:** Gesundheitssystem, Traumazentren, Zertifizierung, Kompensierte Krisenversorgung, Körperhöhlenverletzungen, Healthcare system, Trauma centers, Certification, Compensated crisis care, Body cavity injuries

## Abstract

**Hintergrund:**

Funktionalität und Behandlungskapazität von Krankenhäusern sind entscheidende Komponenten, um die stationäre Behandlung von Patienten in Krisen und Katstrophen sicherzustellen. Die SARS-CoV-2(„severe acute respiratory syndrome coronavirus type 2“)-Pandemie und der Krieg in der Ukraine zeigen dies. Ziel der vorliegenden Untersuchung ist die Erhebung der Behandlungskapazitäten der Krankenhäuser der Traumnetzwerke der Deutschen Gesellschaft für Unfallchirurgie (DGU) unter der Annahme des Vorliegens einer Schadenslage mit einer Vielzahl zu versorgender Traumapatienten.

**Material und Methode:**

Zur Erhebung der aktuellen Behandlungskapazitäten in Abhängigkeit von den Prinzipien und Standards der Versorgung wurden die 622 Krankenhäuser der Traumanetzwerke befragt. Hierzu wurde über die Akademie der Unfallchirurgie (AUC) der DGU und eine elektronische Plattform (SurveyMonkey) ein Fragebogen zur freiwilligen Teilnahme an der Umfrage online gestellt. Die hier dargestellten Daten stellen einen auf die Fragestellung dieser Arbeit fokussierten Auszug der Gesamtdaten dar.

**Ergebnisse:**

An der Umfrage beteiligten sich 252 der 622 im Dezember 2022 zertifizierten Kliniken (40 %). 250 Datensätze waren verwertbar. Zu gleichen Teilen nahmen lokale, regionale und überregionale Traumazentren teil. Durch eine auf das Überleben fokussierte chirurgische Versorgung („tactical abbreviated surgical care“, TASC) könnten in Bezug auf die abgefragten Szenarien die Versorgungskapazitäten in den einzelnen Sichtungskategorien gesteigert werden. Deutlich wurde aber auch, dass die Verfügbarkeit fertigkeitskompetenter Teams zur chirurgischen Versorgung von Körperhöhlenverletzungen nach wie vor eine Herausforderung darstellt.

**Schlussfolgerung:**

Durch die Umfrageergebnisse wird dargestellt, in welchem Umfang aktuell in den Krankenhäusern der DGU-Traumanetzwerke Behandlungskapazitäten für die Versorgung von Verletzten und Verwundeten vorliegen und in welchem Maße diese gesteigert werden können. Hierbei kann ein Massenanfall von Verletzten initial lokal und vorübergehend aufgrund der Dynamik zu einer dekompensierten Krisenversorgung führen. Ziel aller Bemühungen und Vorbereitungen muss es daher sein, die Krankenhäuser dauerhaft zu ertüchtigen, dass dies möglichst zuverlässig vermieden werden kann, und diese Überlegungen in die Krankenhausstrukturreform mit einzubeziehen.

## Abstract (en)

**Background:**

The functionality and treatment capacity of hospitals are decisive components to safeguard the in-hospital treatment of patients in crises and catastrophes. This is shown by the severe acute respiratory syndrome coronavirus type 2 (SARS-CoV-2) pandemic and the war in Ukraine. The aim of this study is the elicitation of treatment capacities of hospitals in the trauma network of the German Society for Trauma Surgery (DGU) assuming a damage situation with a multitude of trauma patients needing treatment.

**Material and method:**

The 622 hospitals in the trauma network were surveyed to elicit the current treatment capacities depending on the principles and standards of treatment. For this purpose, a questionnaire for voluntary participation in the survey was placed online via the Academy of Trauma Surgery (AUC) of the DGU and an electronic platform (SurveyMonkey). The data presented in this article represent an extract of the total data focussed on the issues involved in the study.

**Results:**

A total of 252 of the 622 hospitals certified in December 2022 (40%) participated in the survey and 250 datasets could be utilized. Local, regional and supraregional trauma centers were equally represented. Using a tactical abbreviated surgical control (TASC) focussed on survival, the treatment capacities in the individual triage categories could be increased with respect to the scenarios in question. It was also clear that the availability of teams skilled in the surgical treatment of body cavity injuries still represents a challenge.

**Conclusion:**

The results of the survey demonstrate the extent to which treatment capacities for the care of injured and wounded patients are currently available in the hospitals of the DGU trauma network and to what extent they can be increased. In this way, due the dynamics a mass casualty incident can initially lead locally and temporarily to a decompensated crisis management. The aim of all efforts and preparations must therefore be to durably strengthen hospitals so that this can be avoided as reliably as possible and to include these considerations in the hospital structural reform.

## Hintergrund/Fragestellung

Die medizinische Versorgung der Bevölkerung an Krankenhäusern ist abhängig von deren Funktionalität und Kapazität in Bezug auf den aktuellen Behandlungsbedarf [16, 19]. Beide Komponenten können durch Katastrophen oder Großschadensereignisse grundlegend beeinträchtigt werden. Eine herausragende Bedeutung haben hierbei die Verfügbarkeit von Personal, Material und Raum [11, 16, 19, 20]. In Bezug auf diese Verfügbarkeit, sind die Versorgungslevel „Individual- oder Standardversorgung“, kompensierte Krisenversorgung und dekompensierte Krisenversorgung beschrieben worden [7, 11, 16, 19, 20].

Im Rahmen einer Katastrophe oder Großschadenslage muss es das oberste Ziel sein, die medizinische Versorgung zumindest in einem Stadium der kompensierten Krisenversorgung zu halten. Anerkannte medizinische Standards können in diesem Stadium noch weitestgehend eingehalten werden [7, 11, 16, 19, 20].

Grundsätzlich sind verschiedene Reaktionsmöglichkeiten des Gesundheitssystems denkbar. Mobilisation von Behandlungskapazitäten und Ressourcen (z. B. Material und Personal) in die belasteten Bereiche, Fokussierung der Behandlungsziele und Reduktion auf essenzielle medizinische Standards um den Schadensort, Umverteilung von Patienten und Verzicht auf elektive Eingriffe.

Um dies in einer akuten Schadenslage zeitnah zu erreichen, ist eine umfangreiche Vorbereitung und Planung erforderlich. Sollte es dennoch zu einer dekompensierten Krisenversorgung kommen, sind alle Bemühungen darauf zu richten, das Stadium der kompensierten Versorgung frühestmöglich wieder herzustellen.

Um eine gute Vorbereitung und Planung auf Großschadensereignisse und Katastrophen zu ermöglichen, wäre ein ständig aktualisiertes Lagebild über die Versorgungskapazitäten der Krankenhäuser ein Idealzustand. Dass dies mit erheblichen Schwierigkeiten verbunden ist, hat die SARS-CoV-2(„severe acute respiratory syndrome coronavirus type 2“)-Pandemie gezeigt [9, 14].

Ebenso ist seit dem Krieg in der Ukraine klargeworden, dass langandauernde Schadenslagen mit dem Anfall einer Vielzahl von Verletzten und einer massiven Schädigung der Infrastruktur von den Planungen nicht ausgeschlossen werden darf.

Diese und weitere mögliche Schadenslagen und vor allem die Möglichkeiten zur Reaktion auf einen erheblich gesteigerten Behandlungsbedarf haben wir ausführlich in einem weiteren Artikel zu dem Thema beschrieben [7]. Bei der Versorgung schwerverletzter Patienten rücken die Traumanetzwerke der Deutschen Gesellschaft für Unfallchirurgie (DGU) in den Fokus der Betrachtung. Die hier etablierten Standards und die Inhalte der aktuell überarbeitete S3 Leitlinie „Polytrauma“ definieren den individualmedizinischen Behandlungsstandard des einzelnen Schwerstverletzten in Deutschland. Die Behandlungskapazitäten der in den Traumanetzwerken zertifizierten Kliniken sind auch Gegenstand einer zielgerichteten Planung und Vorbereitung für den Massenanfall von Verletzten und zur Bewältigung einer Großschadenslage [15].

Ziel dieser Arbeit ist es, die Reaktionsmöglichkeiten zur Mobilisation von Behandlungskapazitäten auf die Strukturen der Traumanetzwerkkliniken zu übertragen und mit den regulär vorgehaltenen Versorgungskapazitäten in ein Verhältnis zu setzen. Anhand der aktuellen Umfrage unter den Krankenhäusern der Traumanetzwerke der DGU wird ein Überblick über aktuell mobilisierbare (unfall-)chirurgische Behandlungskapazitäten in Abhängigkeit von der Priorisierung der Behandlung möglich [2, 5, 6, 8, 10]. Diskutiert wird, welche Chancen und Möglichkeiten die Netzwerkstruktur der Traumazentren für die Krisenversorgung und die erforderlichen Behandlungskapazitäten aktuell darstellen.

## Material und Methoden

### Mobilisierbare Behandlungskapazitäten in den Kliniken der Traumanetzwerke

Zur Darstellung der aktuell vorhandenen Kapazitäten, aber vor allem auch zur Abschätzung der Möglichkeiten zur Steigerung der Kapazitäten wurde innerhalb der Traumanetzwerke in Deutschland eine anonymisierte Onlineumfrage durchgeführt. Kontaktiert wurden jeweils die hinterlegten, für die Traumaversorgung verantwortlichen Ärzte. Dadurch wurde sichergestellt, dass jeweils nur ein Datensatz angelegt wurde.

Gefördert durch das Bundesamt für Bevölkerungsschutz und Katastrophenhilfe (BBK) erfolgt derzeit die Erstellung der S2K-Leitlinie „Klinische Katastrophen Medizin Deutschland“ (LeiKliKatMeD). Die Vorbereitung des Projektes beinhaltete unter anderem die Durchführung einer Umfrage zu den Behandlungskapazitäten in den Traumazentren der DGU.

Neben qualitativen Daten zu den vorgehaltenen fachlichen Qualifikationen und Ressourcen wurden belastbare Kenngrößen für mögliche Behandlungskapazitäten im Rahmen von Katastrophen oder Großschadenslagen erhoben. Abgefragt wurde auch, welche Zentren (überregionales Traumazentrum [ÜTZ], regionales Traumazentrum [RTZ], lokales Traumazentrum [LTZ]) in welchem Ausmaß andere Krankenhäuser unterstützen können.

Die Postleitzahl (PLZ) wurde jeweils abgefragt und für die Zuordnung der PLZ-Region verwendet. Auf eine detailliertere Darstellung wurde verzichtet, da dies in Einzelfällen die zugesicherte Anonymität aufgehoben hätte.

### Durchführung der Onlineumfrage

Durch die Akademie der Unfallchirurgie (AUC) der DGU wurde über eine elektronische Plattform (SurveyMonkey) ein Fragebogen zur freiwilligen Teilnahme an der Umfrage online gestellt. Alle verantwortlichen Ärzte (Sprecher der Traumnetzwerke und Leitende Ärzte der Fachabteilung Unfallchirurgie/Orthopädie in den zertifizierten Kliniken) wurden individuell angeschrieben.

Eine Erinnerung erfolgte nach 6 Wochen und insgesamt war der Fragebogen 12 Wochen online verfügbar.

Die Aussagen zu den angegebenen Behandlungskapazitäten sollten als Selbsteinschätzung nach Vor-Ort-Evaluation der Sachlage erfolgen und sich daran orientieren, was die einzelne Klinik im Rahmen einer Schadenslage an Behandlungskapazitäten melden bzw. akut ermöglichen könnte.

Die operative Versorgung kritischer Blutungen in den Körperhöhlen bei penetrierenden Verletzungen erfordert bestimmte Fertigkeiten und kann eine kritische Mangelressource darstellen. Die zunehmende Spezialisierung in den Kliniken und die aktuelle Entwicklung der Weiterbildungskataloge wirkt sich nicht förderlich auf die Verfügbarkeit dieser fertigkeitskompetenten Operationsteams aus, die diese chirurgische Kompetenz zur Versorgung von Verletzungen der Körperhöhlen besitzen (sog. höhlenkompetente Teams). Aus diesem Grunde wurde diese Kapazität zu unterschiedlichen Zeitpunkten abgefragt.

Folgende Punkte und Aspekte wurden weiterhin erfasst:

- regulär vorhandene Bettenzahlen (betrieben und physisch vorhanden),
- operative Versorgungskapazität durch höhlenkompetente Teams,
- Selbsteinschätzung der Bettenkapazitäten in Bezug auf die Sichtungskategorien,
- Behandlungskapazität unter Maßgabe einer katastrophenmedizinischen Versorgung.

Die hier dargestellten Daten stellen einen bezüglich der Fragestellung fokussierten Auszug der Gesamtdaten dar.

Die Daten wurden jeweils auf Normalverteilung mit dem Shapiro-Wilk-Test geprüft und waren nicht normverteilt. Die grafische Darstellung erfolgt in Box-Plots. Angezeigt werden Median, oberes/unteres Quartil, Standardabweichung und Werte außerhalb davon. Bei dieser qualitativen Analyse wurde auf die statistische Untersuchung auf signifikante Unterschiede verzichtet. Zahlenwerte die im Text genannt werden entsprechen den Mittelwerten.

Zum Zeitpunkt der Umfrage waren insgesamt 622 Kliniken als Traumazentren (TZ) zertifiziert. Davon überregionale TZ (ÜTZ) *n* = 105, regionale TZ (RTZ) *n* = 220, lokale TZ (LTZ) *n* = 297.

## Ergebnisse

### Daten zu den aktuellen Versorgungskapazitäten in den Traumanetzwerken

An dieser Umfrage beteiligten sich 252 der 622 im Dezember 2022 zertifizierten Kliniken. 250 Datensätze waren verwertbar, da mehr als 80 % der Fragen beantwortet wurden (40,2 % aller Netzwerkzentren).

**Figure 1:**
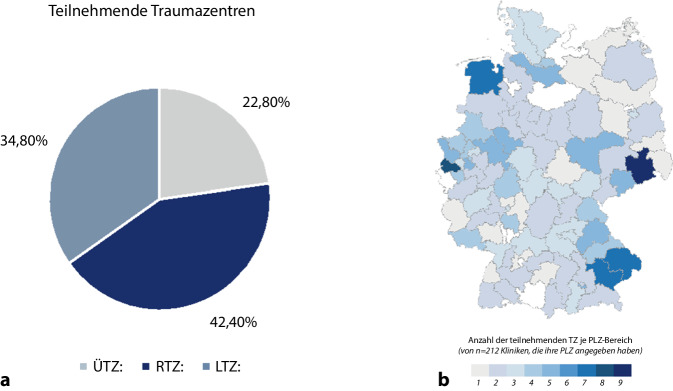

In einem ersten Schritt wurden die Datensätze bezüglich der Lokalisation der teilnehmenden Klinik nach PLZ-Region und Zertifizierungsgrad ausgewertet. Die Abb. 1 zeigt die Ergebnisse.

**Figure 2:**
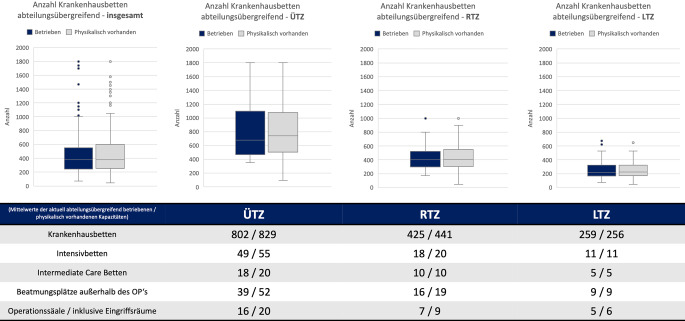

Im nächsten Schritt erfolgte die Auswertung der aktuell physikalisch vorhandenen (strategische Reserve) und aktuell interdisziplinär betriebenen Bettenkapazitäten. Im Mittel betreiben die LTZ 256 von 259 Kranhausbetten, die RTZ 425 von 441 und die ÜTZ 802 von 829. Abb. 2 zeigt die entsprechende Spreizung der Ergebnisse und differenziert noch einmal zwischen Intensiv- bzw. IMC(Intermediate-Care)-Betten und Beatmungsplätzen und Operationssälen.

In einem nächsten Schritt wurde in einer Selbsteinschätzung der Behandlungskapazitäten abgefragt, zu welcher Tageszeit, wie viele Patienten in der vergangenen Woche hätten durch höhlenkompetente Teams notfallmäßig operativ versorgt werden können.

Impliziert wurde hierbei, dass unter Höhlenkompetenz die Fertigkeiten und Fähigkeiten subsumiert werden, einen Patienten mit einer lebensbedrohlichen Blutung in Bauch oder Thorax im Rahmen einer notfallchirurgischen Erstversorgung hämodynamisch zu stabilisieren und die Voraussetzungen für eine erfolgreiche ggf. sekundäre Rekonstruktion ohne Folgeschäden zu etablieren.

**Figure 3:**
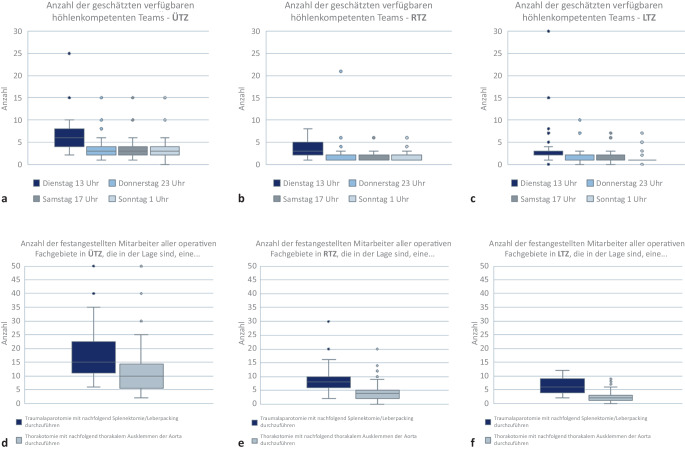

Es zeigt sich, dass die Verfügbarkeit höhlenkompetenter Teams in den ÜTZ außerhalb der regulären Dienstzeit im Mittelwert mit 4 doppelt so hoch ist wie in den RTZ (*n* = 2). In den LTZ ist nachts im Mittel 1 höhlenkompetentes Team verfügbar. Während der regulären Dienstzeit sind es im Mittel in den ÜTZ 7, bei den RTZ 4 und in den LTZ 3 (Abb. 3).

Hierbei ist die Selbsteinschätzung bezüglich der chirurgischen Kompetenz und Erfahrung im Abdomen deutlich höher als im Thorax, dies unabhängig von der Versorgungsstufe des Krankenhauses.

Abschließend wurde die Selbsteinschätzung der Behandlungskapazität nach Sichtungskategorien unter verschiedenen Annahmen abgefragt. Jeweils für die Dauer von 6 und 24 h, mit und ohne Aktivierung des Krankenhausalarm- und -einsatzplanes (KAEP) bzw. unter der Zielsetzung einer individualmedizinischen oder einer auf das Überleben fokussierten taktischen abgekürzten chirurgischen Versorgung („tactical abbreviated surgical care“, TASC).

**Figure 4:**
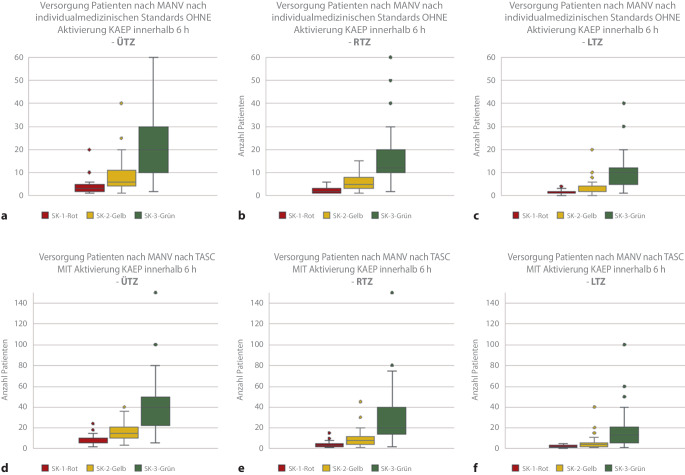

Hier zeigt sich, dass sich die 6‑h-Behandlungskapazitäten durch die Aktivierung des KAEP als auch durch die Fokussierung der Behandlung auf das Überleben möglichst vieler Verletzter (TASC) steigern lassen. Im ersten Fall können in der Sichtungskategorie I Rot im Mittel in einem ÜTZ *n* = 4 Patienten, im RTZ *n* = 2 Patienten und im LTZ *n* = 1 Patient behandelt werden. Nach KAEP-Aktivierung und unter TASC-Zielsetzung schätzen die ÜTZ ihre Behandlungskapazität für SK(Sichtungskategorie)-I-Patienten mit *n* = 8, RTZ *n* = 4 und LTZ *n* = 2 ein (Abb. 4).

**Figure 5:**
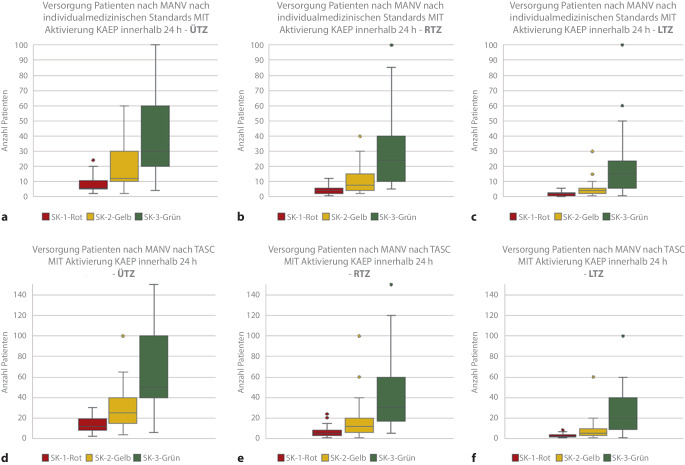

Hierbei zeigen die Abb. 5 und 6, dass die ÜTZ über deutlich größere mobilisierbare Behandlungsreserven verfügen.

**Figure 6:**
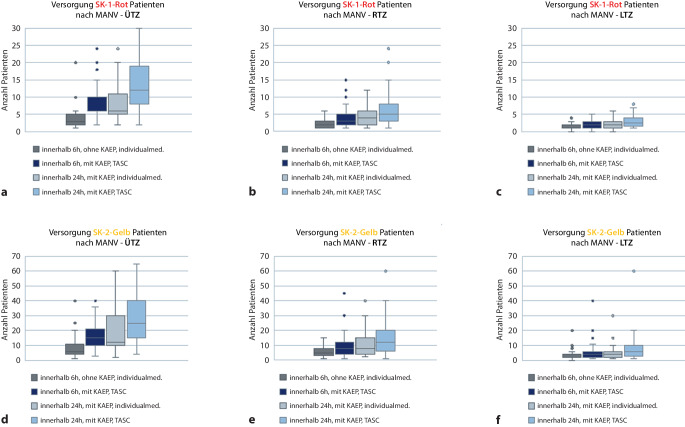

Nach Aktivierung des KAEP und Fokussierung auf TASC-Maßnahmen über einen Zeitraum von 24 h können die Behandlungskapazitäten deutlich erhöht werden (ÜTZ: *n* = 7 SK I und *n* = 16 SK II, RTZ: *n* = 14 SK II und *n* = 29 SK II, LTZ: *n* = 7 SK I und *n* = 16 SK II).

### Berechnung von Gesamtbehandlungskapazitäten und Verteilung der Ressourcen nach Zertifizierung in den Traumanetzwerken der DGU

Legt man die aus der Umfrage errechneten Mittelwerte für die Selbsteinschätzung der Behandlungskapazitäten zugrunde und berücksichtigt die Gesamtanzahl der aktuell in den Traumanetzwerken der DGU® zertifizierten Zentren lässt sich die Lastverteilung in den drei Säulen der Traumnetzwerke bezogen auf eine Schadenslage bzw. die Aufnahmekapazität im Rahmen der katastrophenmedizinischen Krisenversorgung abschätzen.

Für die Berechnung der Tab. 1 wurden die Mittelwerte der gemeldeten Behandlungskapazitäten (Abb. 2) herangezogen und jeweils mit der tagesaktuellen Anzahl der Zentren multipliziert und für die Ermittlung der Gesamtkapazität aufaddiert. Die prozentualen Angaben beziehen sich auf die errechnete Gesamtkapazität.

**Table 1:** 

	Katastrophenmedizinische Krisenversorgung (Physikalisch verfügbare Betten, OP, Eingriffsräume)						
∑ TZ 622 ∑ ÜTZ: 105 ∑ RTZ: 220 ∑ LTZ: 297	∑ ÜTZ	∑ RTZ	∑ LTZ	*∑*	*% ÜTZ*	*% RTZ*	*% LTZ*
Krankenhausbetten	87.045	97.020	76.032	*260.097*	33 %	37 %	29 %
Intensivbetten	5775	4400	3267	*13.442*	43 %	33 %	24 %
IMC-Betten	2100	2200	1485	*5785*	36 %	38 %	26 %
Beatmungsplätze außerhalb des OP	5460	4180	2673	*12.313*	44 %	34 %	22 %
OP	2100	1980	1782	*5862*	36 %	34 %	30 %

*DGU *Deutsche Gesellschaft für Unfallchirurgie, *IMC *Intermediate Care,* LTZ *lokales Traumazentrum, *MANV *Massenanfall von Verletzten, *OP* Operationssaal, *RTZ *regionales Traumazentrum, *SK* Sichtungskategorie,* ÜTZ *überregionales Traumazentrum

Bezogen auf die physikalisch verfügbaren interdisziplinären Behandlungskapazitäten für eine Krisenversorgung repräsentieren die in den Traumanetzwerken zertifizierten Kliniken insgesamt eine Viertelmillion Krankenhausbetten. Hierbei leisten alle Versorgungsstufen jeweils orientierend 25–30 % der Gesamtkapazität.

Wie stellt sich die akute Versorgungskapazität bezüglich der Aufnahme von SK I und SK II Patienten in Abhängigkeit vom Schweregrad des Szenarios dar?

Um diese Frage abschließend ansatzweise beantworten zu können, wurden die in der Befragung dargestellten Bedingungen unter denen das Krankenhaus agieren muss, den Begriffen gesicherte kompensierte, gefährdete kompensierte und dekompensierte Krisenversorgung zugeordnet [7].

**Figure 7:**
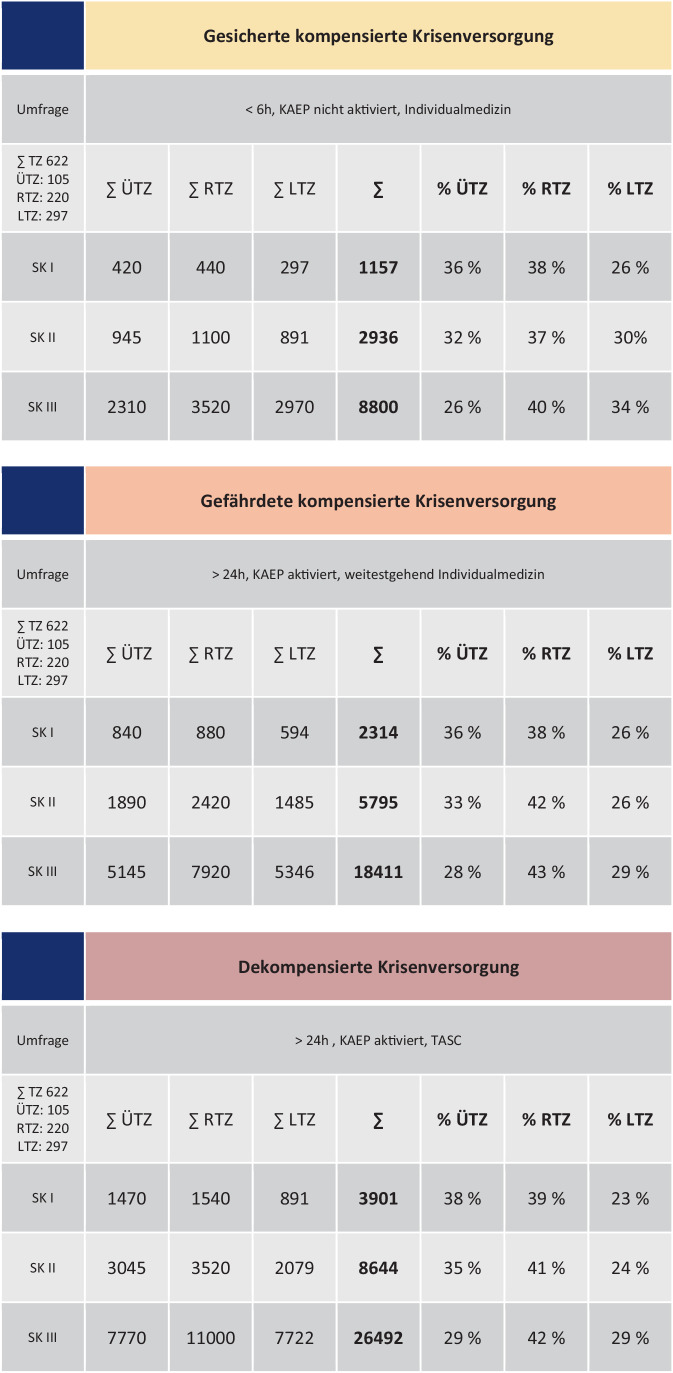

Die Abb. 7 gibt einen Überblick über die unter diesen Annahmen erwartbaren, auf Basis der Mittelwerte der im Rahmen der Selbsteinschätzung (Abb. 4, 5 und 6) genannten Behandlungskapazitäten bezüglich der einzelnen Sichtungskategorien in den Zentren der Traumanetzwerke.

## Diskussion

In der vorliegenden Arbeit wurden die katastrophenmedizinischen und chirurgischen Behandlungskapazitäten in den Kliniken der DGU®-Traumanetzwerke abgefragt. Mit 250 verwertbaren Datensätzen bei 622 zertifizierten Netzwerkkliniken, verteilt über ganz Deutschland und zu annähernd gleichen Teilen bezüglich der Zertifizierung nach ÜTZ, RTZ und LTZ, kann ein aussagekräftiges Abbild der aktuellen Situation skizziert werden.

Die aktuell Diskussion um den dokumentierten Fachkräftemangel im Gesundheitsbereich ist allgegenwärtig [1]. Da ist es auffällig, dass die maximal physikalisch vorhandenen Ressourcen (Bettenanzahl) und die aktuell tagesaktuell betriebenen Ressourcen sich kaum in der Menge unterscheiden. Somit ist davon auszugehen, dass nur eine kleine strategische Reserve physikalisch ungenutzter Behandlungskapazitäten in einem Schadensszenario mobilisierbar wäre.

Bezogen auf die Gesamtbehandlungskapazität in allen Bereichen (Krankenhaus‑, Intensiv- und IMC-Betten) tragen alle Versorgungsebenen (LTZ, RTZ, ÜTZ) zu jeweils ca. 30 % der Gesamtversorgungskapazität bei. Dies kann über die anteilige absolute Anzahl der Klinken ausreichend erklärt werden.

In einem nächsten Schritt wurde die chirurgische Versorgungskapazität anhand der Verfügbarkeit fertigkeitskompetenter Teams für die notfallchirurgische Stabilisierung von Verletzten mit Körperhöhlenblutungen evaluiert.

Qualitativ ist augenscheinlich, dass die überschaubare Verfügbarkeit sog. „höhlenkompetenter Teams“ in allen Versorgungsstufen der Krankenhäuser in den Traumanetzwerken die zeitkritische chirurgische Versorgung von Körperhöhlenverletzungen und die lokale lebensrettende chirurgische Blutstillung in einer Schadenslage limitiert. Hier können Kursformate wie der ACT(Acute Care in Trauma)-Kurs, der chirurgische Fertigkeiten in der Traumaversorgung von Höhlenverletzungen vermittelt, genutzt werden, um die Anzahl höhlenkompetenter Teams zu erhöhen [8].

Zur Kapazitätssteigerung in der temporären Mangelsituation eines kurzfristigen Massenanfalls (z. B. bis zu 6 h) oder einer Schadenslage (bis zu 24 h) stehen die Aktivierung des KAEP sowie auch alternative taktisch-strategische und chirurgische Behandlungskonzepte zur Verfügung.

Für die Darstellung und Bewertung einer Schadenslage unter diesen Gesichtspunkten wurde die einheitliche Nomenklatur der unterschiedlichsten Versorgungsmöglichkeiten von der individualmedizinischen Standardversorgung, über die ungefährdete und gefährdete kompensierte Krisenversorgung bis zur dekompensierten Krisenversorgung mehrfach beschrieben [11, 17, 18]. Die abgefragten Behandlungskapazitäten wurden unter Berücksichtigung der unterschiedlichen Konzepte und Annahmen erhoben. Hierbei entspricht die kurzfristige Versorgung (< 6 h) nach den Maßstäben einer Standardversorgung am ehesten der Situation einer gesicherten kompensierten Krisenversorgung. Die Bewältigung einer Schadenslage über einen Zeitraum von 24 h mit Aktivierung des Krankenhausalarm- und -einsatzplanes (KAEP) zur Sicherstellung einer medizinischen Behandlung nach individual medizinischen Gesichtspunkten, aber ohne Elektivversorgung beschreibt den Zustand einer gefährdeten kompensierten Krisenversorgung. Der Zustand einer dekompensierten Krisenversorgung wurde in der Befragung mit der Zielsetzung, allen Patienten ein Überleben zu ermöglichen (TASC), und Aktivierung des KAEP über einen Zeitraum von 24 h verbunden.

Die erhobenen Daten können somit nur für eine orientierende Abschätzung der Behandlungskapazität im Rahmen von Katastrophen und Großschadenslagen herangezogen werden. Es zeigte sich für die dekompensierte Krisenversorgung, dass die Hauptbelastung aufgrund der Gesamtanzahl von den RTZ übernommen wird. Die LTZ können zwischen 20 und 34 % der Patienten aufnehmen. Je nach Kategorie und Szenario wird theoretisch jede Säule mit einem Viertel bis ca. einem Drittel aller Patienten belastet.

Trotz aktueller Ideen, Konzepte und Tendenzen, die individualmedizinische Schwerstverletztenversorgung einzelner Patienten auf z. B. ÜTZ zu konzentrieren, muss festgestellt werden, dass alle Kliniken der Traumanetzwerke, insbesondere die LTZ, in der flächendeckenden katastrophenmedizinischen Krisenversorgung einer Schadenslage einen berechtigten Stellenwert haben. Dies muss bei der Weiterentwicklung der Krankenhausstrukturreform berücksichtigt werden, da sonst etablierte Behandlungskapazitäten für die Versorgung einer Katastrophen oder Großschadenslage wegfallen.

Auch wenn die lokalen Traumazentren bezüglich der akuten Behandlungskapazität nur geringere Mengen vorhalten können, sind sie für die erste Versorgung einer Schadenslage in der Peripherie, die chirurgische Stabilisierung und Verteilung von Patienten ein unabdingbarer Beitrag. Die LTZ stellen darüber hinaus als lokaler Endversorger, genauso wie in der arbeitstäglichen qualitativ hochwertigen chirurgischen Versorgung von einfachen und der Notfallerstversorgung komplexer Monoverletzungen, eine wichtige Stütze der flächendeckenden Traumaversorgung dar.

In der Selbsteinschätzung der Behandlungskapazitäten nach Sichtungskategorien konnte gezeigt werden, dass die Aktivierung des KAEP und die Fokussierung der Notfallversorgung auf eine taktisch abgekürzte chirurgische Versorgung (TASC) mit Zielsetzung „Überleben jedes Patienten“ die Anzahl der behandelbaren Patienten nach Sichtungskategorien verdoppelt [4–6].

Dies ist ein wichtiger Aspekt und belegt den Stellenwert und die Wichtigkeit der Eingangssichtung (ex ante). Entsprechend dieser Kategorisierung (Eingangssichtung) ist nachfolgend die Priorisierung (Art und Reihung der chirurgischen Notfallinterventionen und -operationen) als auch die Disposition (weitere Diagnostik und Ressourcenzuteilung) und abschließende Realisierung der Notfallversorgung im Rahmen der dekompensierten Krisenversorgung erfolgreich abbildbar. Hierfür gibt es etablierte Versorgungskonzepte [3, 5, 8].

Durch die Befragung der Krankenhäuser der Traumanetzwerke konnte gezeigt werden, dass die Begrifflichkeiten einer abgestuften taktischen medizinischen und chirurgischen Versorgung (TASC) bei den teilnehmenden verantwortlichen Ärzten der Kliniken in den Traumanetzwerken vorhanden sind. Hierbei zeigt sich, dass durch die Änderung der Zielsetzung der Behandlung auf eine taktische Versorgung hin zusätzliche Behandlungskapazitäten vor Ort realisiert werden können.

Die Anpassung und stetige Verbesserung des KAEP unter diesen Aspekten, die fachliche Begleitung und Entwicklung katastrophenmedizinischer Kompetenzen, die Vertiefung von Weiterbildungsinhalten und Vermittlung notfallchirurgischer Fertigkeiten durch entsprechende Kursformate (z. B. DSTC[Definitive Surgical Trauma Care]- oder ACT-Kurs, ATLS[Advanced Trauma Life Support]- und TDSC[Terror and Disaster Surgical Care]-Kursen) müssen hier durch Optimierung der Abläufe und Priorisierung weitere Behandlungskapazitäten freisetzen und qualitativ die Versorgung verbessern [2, 4–6, 8].

Übergeordnetes Ziel zur Bewältigung einer Schadenslage in einem entwickelten Gesundheitssystem sollte es immer sein, eine individualmedizinische Versorgung längstmöglich zu gewährleisten bzw. nur kurzfristig zu verlassen und durch alle Maßnahmen dann frühestmöglich wieder herzustellen. Daher ist es eine Kernforderung, die Vorbereitung auf eine Schadenslage a priori nicht auf die mögliche Reduktion der Standards zu limitieren.

Durch die Vorbereitung auf Großschadensereignisse und Katastrophen wird es ermöglicht, genau dieses zu verhindern, die notfallchirurgischen Kapazitäten und Fertigkeiten verfügbar zu machen und auszubauen! Eine taktisch-strategische Versorgung (TASC) mit initial dem Ermöglichen des Überlebens aller in möglichst großer Anzahl kann hierbei nicht als Standard, sondern nur als vorübergehende Zielsetzung der Behandlung gelten.

Zusammen mit möglichen Kompensationsmechanismen (z. B. Verlegung von Patienten, Zufuhr und Mobilisation von Ressourcen durch Aktivierung des KAEP) zur frühestmöglichen Begrenzung der akuten Mangelsituation und Wiedererreichen mindestens der ungefährdeten Krisenversorgung dient alles dem gemeinsamen übergeordneten Ziel, die bestmögliche chirurgische Versorgung für möglichst alle Patienten flächendeckend zu jedem Zeitpunkt zu realisieren.

Bei diesen Bemühungen können die etablierten Strukturen der Traumanetzwerke durch Vorbereitung und einheitliche Konzepte in der medizinischen Krisenversorgung einer Katastrophe oder einer Großschadenslage weiterentwickelt und optimiert werden [12]. Durch Erweiterung von Kooperationsvereinbarungen zum Personalaustausch, gemeinsame Übungen und das Vorhalten von Material können hier gemeinsam verfügbare Behandlungskapazitäten in Netzwerkstrukturen etabliert und ggf. akut mobilisiert werden [13, 21].

Ein erster Schritt ist hier mit der durchgeführten orientierenden Kapazitätsanalyse der Behandlungsmöglichkeiten in den Krankenhäusern der Traumanetzwerke erfolgt. Die Notwendigkeit der Vorbereitung auf katastrophenmedizinische Schadenslagen oder einen Massenanfall von Verletzten kommt auch in der aktuellen Fassung des Weissbuches zur Traumaversorgung der DGU zum Ausdruck und wird weiter in der Neufassung vertieft werden, um der gesamtgesellschaftlichen Verantwortung der Steigerung der Resilienz des Gesundheitssystems für Katastrophen- und Großschadenslagen gerecht zu werden.

Durch z. B. Kostenübernahmen für Maßnahmen und Kurse, die nachweislich der Verbesserung der katastrophenmedizinischen Krisenversorgung dienen, könnten Krankenhäuser bei ihren Bemühungen unterstützt werden. Die bisherigen unter der Zielrichtung auf die Optimierung der Schwerstverletztenversorgung einzelnen ausgestalteten Kooperationsverträge in den Traumanetzwerken kann man dahingehend weiterentwickeln, dass z. B. jederzeit unbürokratisch und niedrigschwellig eine Umverteilung von Personal, Material und Patienten zur Bewältigung einer Schadenslage erfolgen kann.

Die Definition relevanter Inhalte für die Qualifizierung, die Etablierung und den Erhalt notfallchirurgischer Fertigkeiten bzw. den Kompetenzerhalt der an der katastrophenmedizinischen Krisenversorgung beteiligten Kliniken könnte z. B. durch eine gemeinsame, die Interessen von Bund und Ländern beim gesundheitlichen Bevölkerungsschutz bei der Bewältigung einer Schadenslage vertretende zentrale einzurichtende Institution (vergleichbar z. B. dem Havariekommando der Küstenländer und des Bundes) erfolgen.

Gleichzeitig erscheint es zielführend, dass auch die Erfordernisse der Versorgung von Katastrophen- und Großschadenslagen in die Überlegungen zur Krankenhaustrukturreform miteinbezogen werden. Im Umkehrschluss sind alle Weiterentwicklungen unseres Gesundheitssystems dahingehend zu prüfen, ob sie sich nachteilig auf eine flächendeckende Bewältigung eines Massenanfalls oder einer Schadenslage auswirken. Gemeinsame von Bund und Ländern verfolgte katastrophenmedizinische Standards können die Basis sein für weitergehende Überlegungen und eine langfristige Entwicklung der Krankenhausstruktur.

## Schlussfolgerung

Durch die Umfrageergebnisse konnte gezeigt werden, in welchem Umfang aktuell in den Krankenhäusern der Traumanetzwerke katastrophenmedizinische und chirurgische Behandlungskapazitäten vorliegen.

Hierbei kann eine Katastrophen- oder Großschadenslage mit Massenanfall von Verletzten oder Erkrankten je nach Ausmaß initial lokal und vorübergehend aufgrund der Dynamik zu einer dekompensierten Krisenversorgung führen. Hier sind die Behandlungskapazitäten begrenzt. Umverteilung von Patienten, akute Mobilisation von Personal, Material und die Fokussierung der chirurgischen Versorgung (TASC) kann die Behandlungskapazitäten kurzfristig steigern.

Es ist daher wichtig, die aktuell zertifizierten Zentren der Traumanetzwerke und die noch nicht zertifizierten oder an einem Traumanetzwerk teilnehmenden Krankenhäuser bei ihren Bemühungen zu unterstützen, die erreichten Qualitätsstandards der Traumaversorgung in Krisensituationen aufrecht zu erhalten und die Vorbereitung auf solche Ereignisse zu intensivieren. Nur so lässt sich das Ziel der möglichst langen Aufrechterhaltung einer kompensierten Krisenversorgung oder einer schnellstmöglichen Rekompensation erreichen.

Aus Sicht der DGU und um die notfallchirurgische Versorgung in Deutschland zu stärken, muss daran gearbeitet werden, die Zentren der Traumanetzwerke auch unter katastrophenmedizinischen Aspekten weiter zu qualifizieren, um der gesellschaftlichen Verantwortung einer flächendeckenden chirurgischen Trauma- und katastrophenmedizinischen Versorgung dauerhaft und durchhaltefähig gerecht zu werden.
